# First Trimester of Pregnancy as the Sensitive Period for the Association between Prenatal Mosquito Coil Smoke Exposure and Preterm Birth

**DOI:** 10.3390/ijerph191811771

**Published:** 2022-09-18

**Authors:** Xin-Chen Liu, Esben Strodl, Li-Hua Huang, Qing Lu, Yang Liang, Wei-Qing Chen

**Affiliations:** 1 Department of Epidemiology, School of Public Health, Sun Yat-sen University, Guangzhou 510080, China; 2 School of Psychology and Counselling, Queensland University of Technology, Brisbane, QLD 4000, Australia; 3 School of Health, Xinhua College of Guangzhou, Guangzhou 510080, China

**Keywords:** mosquito coil smoke, prenatal exposure, preterm birth, sensitive period, child sex

## Abstract

Mosquito coils are efficient mosquito repellents and mosquito coil smoke (MCS) contributes to indoor air pollution. However, no prior population-based study has investigated whether prenatal MCS exposure is a risk factor for preterm birth (PTB) and whether exposure to MCS in different trimesters of pregnancy is associated with different levels of risk. The sample involved 66,503 mother–child dyads. Logistic regression models were used to examine the relationships between prenatal MCS exposure during different trimesters of pregnancy and PTB. We found that prenatal MCS exposure was associated with a greater likelihood of PTB (OR = 1.12, 95%CI: 1.05–1.20). The prenatal MCS exposure during the first trimester was associated with 1.17 (95%CI: 1.09–1.25) times the odds of being PTB, which was higher than exposure during the second trimester (OR = 1.11, 95%CI: 1.03–1.19) and during the third trimester (OR = 1.08, 95%CI: 1.01–1.16). In the stratified analysis, prenatal MCS exposure significantly increased PTB risk among girls but not among boys. Our results indicated that maternal MCS exposure during pregnancy was associated with PTB and that the first trimester might be the sensitive period. In light of these findings, public health interventions are needed to reduce prenatal exposure to MCS, particularly during the first trimester of pregnancy.

## 1. Introduction

Preterm birth (PTB) is defined as infants born alive before 37 weeks’ gestational age. The WHO reported that, globally, an estimated 15 million babies are born preterm every year, and the rate is increasing in almost all countries [1]. The 10 countries with the greatest number of preterm births are mainly low- to middle-income countries (LMICs) from southeastern Asia, South Asia, South America and Africa [1]. PTB is the leading cause, and the second most common cause, of death in children under the age of 5 years worldwide and in China, respectively [2,3]. It is also associated with long-term consequences such as adverse neurodevelopmental diseases and chronic noncommunicable diseases later in life [4,5]. Its short-term and long-term consequences have placed a heavy burden on both individual families and society [6].

Mosquito coils are widely used efficient mosquito repellents, and mosquito coil smoke (MCS) is a common source of indoor air pollution in these above-mentioned LMICs [7]. The mostly widely used active ingredients of the mosquito coil are pyrethrin/pyrethroids [8,9]. They are widely used because they were originally believed to have low toxicity in humans and untargeted animals [8]. However, recently, emerging studies show that they may cause a variety of toxicities in humans, including having adverse effects on reproductive health [10]. In addition to the active ingredients, mosquito coils include dyes, binders, organic fillers and other additives capable of smoldering well. However, the smoldering of mosquito coils releases indoor air pollutants such as particulate matter (PM), violative organic compounds (VOCs) and trace metals generated from the remaining components of the mosquito coil [7]. Both the pyrethrin/pyrethroids and the indoor air pollutants evaporated with the smoldering of mosquito coils might have detrimental effects on health [8].

In the past two decades, scientists have largely investigated the adverse effects of exposure to MCS on lung cancer and respiratory health in population-based studies [11,12,13]. To the best of our knowledge, there has been no prior study investigating the associations of maternal MCS exposure during pregnancy with PTB. Moreover, no prior study has investigated the sensitive period during pregnancy for MCS exposure. The sensitive period, in life course epidemiology, refers to a specific exposure period that has a stronger impact on disease onset and development than other exposure periods [14]. To date, the existence of a sensitive period for this association remains unknown and so this study aimed to investigate whether different trimesters of pregnancy may be sensitive periods for MCS exposure being a risk factor for PTB.

## 2. Materials and Methods

### 2.1. Study Population

Data from the 2021 survey of Longhua Child Cohort Study (LCCS) was used for this study. This survey was conducted among 235 kindergartens in the Longhua District of Shenzhen, China, with 69,638 mother–child dyads being enrolled. After excluding mothers (1) who were active smokers (n = 1314), (2) whose child (the child involved in this study) was not a singleton birth (n = 1795), (3) who did not report their MCS exposure status during pregnancy (n = 2) and (4) those who did not report whether or not their child had a hospital based PTB diagnosis (n = 24), a total of 66,503 (95.49%) mother–child dyads were included for the analyses (Figure S1). Within this sample, 5277 questionnaires lacked information on at least one selected covariate. Since multiple imputation (MI) is recommended for managing missing data in environmental epidemiology research [15], we used MI to impute the missing covariates in the statistical analyses performed in this study. All participants provided written informed consent during enrolment. The study was approved by the Ethic Committee of the School of Public Health of Sun Yat-sen University.

### 2.2. Data Collection

An online self-administered structured questionnaire was distributed to the mothers of children attending one of the designated kindergartens. If required, the mothers could ask for guidance from well-trained interviewers on how to complete the questionnaire. Information about the demographic characteristics of both parents and children, the medical history and pregnancy complications experienced by the mothers, maternal household air pollution exposure during pregnancy and whether or not PTB was diagnosed by a doctor were collected in the questionnaire.

#### 2.2.1. Prenatal MCS Exposure Measurement

First, prenatal MCS exposure during the entire pregnancy was measured by asking mothers the following question: “Did you and/or your family members burn mosquito coils at home during your pregnancy?” (Answer was ‘NO’ or ‘YES’.) Mothers who reported ‘YES’, we defined them as the MCS exposure group, while those who reported ‘NO’ were defined as the reference group.

Second, in order to distinguish the sensitive period for association between prenatal MCS exposure and PTB, MCS exposure in each of the three trimesters of pregnancy was measured by asking: (1) “Did you and/or your family members burn mosquito coils at home during your first trimester (1–13 weeks) of pregnancy?” (Answer was ‘NO’ or ‘YES’.) (2) “Did you and/or your family members burn mosquito coils at home during your second trimester (14–27 weeks) of pregnancy?” (Answer was ‘NO’ or ‘YES’.) (3) “Did you and/or your family members burn mosquito coils at home during your third trimester (28 weeks to delivery) of pregnancy?” (Answer was ‘NO’ or ‘YES’.) In each trimester, mothers who reported ‘YES’ were defined as the MCS exposure group, and the participants who reported ‘NO’ were considered the reference group. We conducted a cross-over analysis to further verify the sensitive period we found. This was performed by dividing the participants into 8 subgroups according to different combinations of MCS exposure status (No or Yes) in each trimester. Mothers who had never been exposed to MCS during the entire pregnancy were categorized into subgroup 1. Mothers who were exposed to MSC only during the first, second or third trimester were categorized into subgroups 2, 3 and 4, respectively. Subgroups 5, 6 and 7 consisted of mothers who were exposed in the first and second, second and third and first and third trimesters, respectively. Finally, subgroup 8 included mothers who were exposed to MCS during all three trimesters of pregnancy.

#### 2.2.2. Preterm Birth Assessment

PTB status was determined by the mothers’ response to the following question: “Whether the child involved in this study was diagnosed with PTB at birth by a doctor?” (Answer was ‘NO’ or ‘YES’.)

#### 2.2.3. Covariates Collection

Based on existing studies and data accessibility [16,17,18,19,20,21,22], certain parental and fetal risk factors for PTB were initially included: maternal age at conception, maternal education, marital status, household income, frequency of prenatal care visit, pre-pregnancy BMI, parity, certain pregnancy complications such as pregnancy induced hypertension (PIH), pre-eclampsia (PE) and gestational diabetes mellitus (GDM) and child’s sex and birth season. In addition, exposure to other sources of indoor air pollution has been shown to be associated with PTB [16,23,24,25] and so were also included. Then, a directed acyclic graph (DAG, Table S1) was constructed to select a minimal sufficient set of covariates (DAGitty v3.0 software, http://www.dagitty.net (accessed on 21 January 2021)). There is also evidence that prenatal air pollution exposure increases the risk of some pregnancy complications, including PIH, PE and GDM [26,27], and that they also may be risk factors for PTB [28,29]. As such, they might represent mediating variables on the pathway between prenatal MCS exposure and PTB. According to this reason and the results of DAG, we excluded them from our selected covariates. Maternal age in years was considered as a continuous variable. Maternal education was categorized into three groups (less than high school, high school and greater than high school). Monthly household income was categorized into three groups (<RMB 20,000, RMB 20,000–39,999 and ≥RMB 40,000). Marital status was defined as married or not married (which included single, separated and divorced). Parity was dichotomized as nulliparous and multiparous. Frequency of prenatal care visits was categorized into 3 groups (never, 1–6 times and ≥7 times). Mothers were asked to record in the questionnaire their pre-pregnancy weight (in kilograms) and height (in meters), and pre-pregnancy BMI was calculated and categorized into underweight (<18.5 kg/m^2^), normal weight (18.5–23.9 kg/m^2^) and overweight (>24 kg/m^2^) [30]. In addition, prenatal exposure to four other sources of household air pollution, including environmental tobacco smoke exposure, cooking oil fumes exposure, incense smoke exposure and house renovation exposure were questioned, and each was dichotomized as yes or no. The child’s sex (boy or girl) and birth season (spring, summer, autumn or winter) were also recorded.

### 2.3. Statistical Analyses

We utilized univariate and multivariate logistic regression models to evaluate the association between prenatal MCS exposure and PTB before and after adjusting for the selected potential covariates. This involved first estimating the association between prenatal MCS exposure and risk of PTB during the entire pregnancy. We also estimated the effect of prenatal MCS exposure during different trimesters of pregnancy on the risk of PTB to elucidate which trimester of pregnancy might be the sensitive period for this association. Then, a cross-over analysis was further applied to provide supplementary proof for the sensitive period we found in this association (Table S1).

Since prior studies have reported that the different sex of the fetus might have different responses to environmental exposures [16,31,32,33], we conducted a stratification analysis to test whether our targeted associations varied by child sex. We also conducted a sensitivity analysis to assess whether above associations were robust among 61,226 participants with complete data in all the selected covariates. Statistical analyses were performed with R statistical software (version 4.0.0, http://www.r-project.org (accessed on 6 May 2020)) and the significance level was set at *p* < 0.05.

## 3. Results

### 3.1. Population Characteristics

The characteristics of the study sample are shown in Table 1. The mean age of the mothers was 28.28 years. Over 84% of the mothers had at least high school education, 97.6% were married, 32.6% were nulliparous, 76.4% had over seven times prenatal care visits and about half of the households earned more than RMB 20,000 every month. In addition, 68.2% of the mothers had normal pre-pregnancy BMI. About half of the children were girls and the birth seasons were evenly spread across the four seasons. The prenatal MCS exposure rate was 30.6% and the PTB prevalence was 7.2% in our sample.

### 3.2. Prenatal MCS Exposure in Different Periods of Pregnancy with Risk of PTB: Identifying the Sensitive Period

The associations between prenatal MCS exposure during different periods of pregnancy and PTB are presented in Table 2. Compared with the reference group, maternal MCS exposure during the entire pregnancy was significantly associated with an increased risk of PTB (OR = 1.12 (95%CI: 1.05–1.20)). Moreover, maternal MCS exposure during each trimester of pregnancy were all significantly associated with higher PTB risk compared with the reference group. The prenatal MCS exposure during the first trimester was associated with 1.17 (95%CI: 1.09–1.25) times the odds of being PTB, which was higher than exposure during the second trimester (OR = 1.11, 95%CI: 1.03–1.19) and during the third trimester (OR = 1.08, 95%CI: 1.01–1.16). The cross-over analysis also indicated that MCS exposure only in the first trimester contributed to a significantly higher PTB risk than exposure only in the second or third trimester of pregnancy, and MCS exposure in the first trimester, in both the first and second trimester, as well through all three trimesters of pregnancy significantly increased the risk of PTB (Table S1).

### 3.3. Subgroup and Sensitivity Analysis

We further conducted stratified analyses to examine the association between maternal MCS exposure during different trimesters and PTB risk according to the child’s sex (see Table 3). The risk of PTB was significantly increased among girls but not among boys in each of the pregnancy periods. Among girls, maternal MCS exposure during the entire pregnancy, the first trimester, the second trimester and the third trimester all significantly increased the risk of PTB, with ORs of 1.22 (95%CI: 1.11–1.35), 1.28 (95%CI: 1.16–1.42), 1.22 (95%CI: 1.10–1.35) and 1.19 (95%CI:1.07–1.32), respectively. Among boys, prenatal MCS exposure was not associated with PTB in any of the pregnancy periods. The results of the cross-over analyses examining the trimester-specific association between prenatal MCS exposure and PTB stratified by the child’s sex showed similar results (Table S2).

We also conducted the sensitivity analysis after excluding participants with any missing value on any of the involved covariates (n = 5277). These exclusions had no significant effects on the estimated associations between prenatal MCS exposure and PTB (Tables S3 and S4).

## 4. Discussion

In this study, we found that prenatal MCS exposure during pregnancy increased the risk of PTB. More specifically, mothers exposed during the first trimester of pregnancy had a higher risk of PTB than those exposed during the other two trimesters. When we examined more precisely the timing of MCS exposure, we found that MCS exposure in the first trimester, in both the first and second trimester, as well through all three trimesters of pregnancy significantly increased the risk of PTB. These findings indicate that the first trimester of pregnancy may be a particularly sensitive time for maternal MCS exposure being a risk factor for PTB. In the subgroup analyses, prenatal MCS exposure was a significant risk factor for PTB among girls but not among boys, and the sensitive period was still identified as being during the first trimester for girls.

To date, this is the largest population-based study to find the association between prenatal exposure to MCS and PTB. Burning mosquito coils indoors to repel mosquitoes is common in tropical or subtropical areas, especially in LMICs [34]. Interestingly, more than 60% of PTB occur in these areas and poorer countries are at higher risks [1]. Since mosquito coils are rarely used in Western countries, few studies have shed light on the detrimental effect of prenatal MCS exposure on pregnancy outcomes. Recently, emerging evidence has indicated that the active ingredients contained in MCS might be detrimental to birth outcomes. Although to date no studies have found an association between prenatal pyrethroids exposure and PTB or length of gestation [35,36,37], a prior study conducted in rural northern China reported that prenatal pyrethroid insecticide use was associated with a decrease in birth weight [38]. Another study found a positive association between prenatal pyrethroid exposure and head circumference among boys [17]. Except for pyrethroid, the smoldering of base materials in mosquito coils releases many other pollutants, such as PM, carbon monoxide (CO) and polycyclic aromatic hydrocarbons (PAHs) [7]. There is strong evidence indicating that prenatal exposure to PM_2.5_, PM_1_, PM_10_ or PM_2.5_ constituents increases the risk of PTB [39,40,41,42,43,44]. Studies have also reported that CO and PAHs are potential risk factors for PTB [45,46,47]. Building upon the speculations from these previous studies, this study hypothesized that prenatal exposure to MCS might result in a significantly higher risk of PTB.

David Barker proposed that at different periods of life there are distinct levels of sensitivity to exposure to environmental risk factors [48]. Since then, scientists have begun to explore the effect of time-specific environmental exposure on birth outcomes, in order to identify the sensitive periods for various environmental risk factors. For example, a previous study found phthalate exposure in early pregnancy significantly increased the risk of PTB, but not in middle or late pregnancy [49]. Another study indicated that prenatal PM_2.5_ exposure during the second trimester but not the third trimester was associated with fetal growth characteristics [50]. Cheng and colleagues observed that maternal cadmium exposure was inversely associated with fetal growth only during the first trimester of pregnancy [51]. Unfortunately, there has been a paucity of studies examining the time-specific effect of prenatal MCS exposure on the risk of PTB. Interestingly, our study found prenatal MCS exposure in the first trimester of pregnancy was associated with a higher risk of PTB than in the other two trimesters. Moreover, the cross-over analyses further indicated that maternal MCS exposure only in the first trimester contributed to a significantly higher PTB risk than exposure only in the second or third trimester of pregnancy. Accordingly, we hypothesized that the first trimester of pregnancy might be the sensitive period linking prenatal MCS exposure and PTB. However, the results of the cross-over analysis might be insufficiently powered because of the small sample sizes in several subgroups and so requires replication in future studies.

A number of possible biological mechanisms may help to explain why the first trimester could be the sensitive period for the association between prenatal MCS exposure and PTB. Since the placenta establishes a connection between the mother and the fetus, the healthy growth of the fetus is critically dependent on normal placental function, which is dependent on the normal invasion of trophoblast and sufficient blood circulation in the utero-placental system [52,53]. The burning of one mosquito coil has been shown to release the same amount of particular matters as burning 75–137 cigarettes, and the amount of formaldehyde as burning 51 cigarettes [11]. Furthermore, the continuous smoldering of a mosquito coil at night will emit CO that exceeds the WHO statutory limit of 9.0 ppm for indoor environments [54]. Exposure to air pollutants contained in mosquito coil smoke may cause maternal systematic inflammation and oxidative stress [55,56,57]. In addition, the active ingredients of mosquito coils may provoke apoptosis, lipid, protein and DNA damage along with toxic effects through oxidative stress, reactive oxygen species (ROS) and reactive nitrogen species (RNS) [58]. The timing of the maternal exposure to MCS is important, given that it is acknowledged that the first trimester of pregnancy is critical for placenta development [59,60,61]. The inflammatory response and oxidative environment caused by first trimester MCS exposure may result in poor placentation and inadequate uterine artery transformation and remodeling [62,63], which can contribute to placental dysfunction, characterized by impaired uteroplacental perfusion, chronic hypoxia and placental ischemia and finally can lead to PTB [64,65,66]. Thus, there appears to be plausible biological processes that may contribute to the first trimester of pregnancy being the sensitive period for prenatal MCS exposure causing PTB through placental dysfunction.

In the stratified analysis, the results indicated that prenatal MCS exposure was significantly associated with PTB risk among female infants, but not among male infants. This sex difference is consistent with some previous studies examining the association between prenatal environmental exposure and pregnancy outcomes. Lee and colleagues found the association between prenatal bisphenol A exposure and birth weight varied by fetal sex [67]. A retrospective study indicated that prenatal stress was significantly associated with PTB and LBW in girls but not in boys [68]. Another prospective study reported that prenatal cadmium exposure was associated with a decrease of birthweight in girls but not in boys [69]. Regarding household air pollution, a retrospective study found that paternal smoking exposure and home renovation exposure during pregnancy was significantly associated with PTB in boys but not in girls, while pet-keeping during gestation was significantly associated with LBW and term LBW in girls but not in boys [16]. In addition, a recent prospective birth cohort study indicated that the household use of wood as a primary cooking fuel was associated with SGA only among girls [33].These findings all suggest that child sex might moderate the associations between prenatal environmental exposure and pregnancy outcomes. Some studies have indicated that different placental lesions and adaptations between male and female infants might be the potential biological mechanisms underlying theses sex differences in birth outcomes [31,70,71]. However, some studies have proposed that if conditions during pregnancy are stressful, then male fetuses will preferentially be culled compared with female fetuses, which means this sex-specific association might be influenced by the differential “culling” of fetuses by sex [72,73]. Thus, we should be careful when we interpret our results that prenatal MCS exposure increase the risk of PTB among girls but not among boys and more studies are needed to confirm this finding.

As far as we know, this large study of 66,503 mother–child pairs is the first study to examine and identify the sensitive period for the association of prenatal MCS exposure and PTB. However, our study had some limitations that should be considered when interpreting the results. First, we excluded a small number of participants (n/N = 26/69,638) because of their missing values in either the MCS exposure status or the PTB diagnosis. If these data were missing completely at random (MCAR), then the study sample was a random sampling of the enrolled population. If these data were missing not at random (NMAR), then they might introduce bias when interpreting the results of our study. However, considering the proportion of missingness is very small (about 0.04%), even if the data were NMAR, it would not introduce a large bias. Second, MCS exposure during pregnancy was retrospectively self-reported by mothers. While it is possible that there may have been some recall bias regarding the exact timing of trimesters, we attempted to minimize this by ensuring that 96% of the participants received handbooks that specified the timelines for each trimester at their first prenatal care visit. Nonetheless, it is possible that there may still have been some misclassification of trimesters by the participants and that these misclassifications might have influenced the strength of the true relationship between prenatal MCS exposure in each trimester and PTB in our results. Third, PTB was assessed through mothers’ recall of hospital diagnosis. Although this methodology of utilizing self-reported measures of birth outcomes is of great value in conducting a preliminary large population study [16,23], there is a risk of recall bias affecting the findings. Fourth, the frequency of MCS exposure, and specific air pollutants exposed to, in the three trimesters were not measured in this study. As such, we could only identify, but not quantify further, the detrimental effect of prenatal MCS exposure during different periods of pregnancy on PTB. Fifth, although a range of variables were included as covariates, the list of covariates was not comprehensive. For example, in this study we did not record mothers’ previous history of PTB, their neighborhood socioeconomic or environmental conditions, their household ventilation condition and dietary intake, as well as their ambient air pollution exposure during pregnancy, all of which could also be potential covariates and should be considered in future research designs. Finally, retrospective studies provide weaker evidence than prospective studies in confirming the direction of causal inferences and in testing biological explanations for the associations identified. Future prospective cohort studies using individual air pollutant monitors and collecting both biological specimens and self-report questionnaires in every trimester of pregnancy are needed to replicate these results.

## 5. Conclusions

To conclude, this study indicated that prenatal MCS exposure increased the risk of PTB and the first trimester of pregnancy might be the sensitive period. In light of these findings, public health interventions are needed, especially in LMICs, to reduce prenatal exposure to MCS in the first trimester of pregnancy. This may be particularly important when the fetus is female. Further prospective studies are needed to replicate this important finding.

## Figures and Tables

**Table 1:** Characteristics of the study participants (n = 66,503).

Characteristics	Mean ± SD or n (%)
Maternal age at conception, years, Mean ± SD	28.28 ± 4.40
Maternal education, n (%)	
Less than high school	10,054 (15.1)
High school	13,590 (20.4)
Greater than high school	42,859 (64.4)
Household income, RMB/month, n (%)	
<20,000	33,196 (49.9)
20,000–39,999	22,231 (33.4)
≥40,000	11,076 (16.7)
Marital status, n (%)	
Married	64,888 (97.6)
Not married	1615 (2.4)
Parity, n (%)	
Nulliparous	21,659 (32.6)
Multiparous	44,844 (67.4)
Frequency of prenatal care visits, n (%)	
0	3919 (5.9)
1–6	11,772 (17.7)
≥7	50,812 (76.4)
Maternal pre-pregnancy BMI, kg/m^2^, n (%)	
<18.5	13,269 (20.0)
18.5–23.9	45,369 (68.2)
>24	7865 (11.8)
Child’s sex, n (%)	
Boy	35,507 (53.4)
Girl	30,996 (46.6)
Birth season, n (%)	
Spring	15,523 (23.3)
Summer	16,236 (24.4)
Autumn	18,205 (27.4)
Winter	16,539 (24.9)
MCS exposure, n (%)	
No	46,167 (69.4)
Yes	20,336 (30.6)
PTB, n (%)	
No	61,716 (92.8)
Yes	4787 (7.2)

PTB—preterm birth; BMI—body mass index; MCS—mosquito coil smoke.

**Table 2:** Associations between maternal MCS exposure during different periods of pregnancy and PTB.

Exposure Periods	MCS Exposure	PTB/n	ORs (95% CI)	
			Crude	Adjusted ^a^
Entire pregnancy	No	3186/46,167	1.00	1.00
	Yes	1601/20,336	1.15 (1.08, 1.23)	1.12 (1.05, 1.20)
The first trimester	No	3340/48,643	1.00	1.00
	Yes	1447/17,860	1.20 (1.12, 1.27)	1.17 (1.09, 1.25)
The second trimester	No	3461/49,641	1.00	1.00
	Yes	1326/16,862	1.14 (1.07, 1.22)	1.11 (1.03, 1.19)
The third trimester	No	3548/50,502	1.00	1.00
	Yes	1239/16,001	1.11 (1.04, 1.19)	1.08 (1.01, 1.16)

^a^ Adjustment for maternal age at conception, maternal education, marital status, household income, frequency of prenatal care visits, pre-pregnancy BMI, parity, child’s sex, birth season and exposure to other four sources of household air pollution. Abbreviations: MCS—mosquito coil smoke; PTB—preterm birth.

**Table 3:** Adjusted associations of PTB with maternal MCS exposure during different periods of pregnancy, stratified by the child’s sex ^#^.

Exposure Periods	Child’s Sex	aORs (95% CI)
Entire pregnancy	Boys	1.05 (0.96, 1.15)
	Girls	1.22 (1.11, 1.35)
The first trimester	Boys	1.09 (0.99, 1.19)
	Girls	1.28 (1.16, 1.42)
The second trimester	Boys	1.03 (0.94, 1.13)
	Girls	1.22 (1.10, 1.35)
The third trimester	Boys	1.00 (0.91, 1.10)
	Girls	1.19 (1.07, 1.32)

^#^ Adjustment for maternal age at conception, maternal education, marital status, household income, frequency of prenatal care visits, pre-pregnancy BMI, parity, child’s sex, birth season and exposure to other four sources of household air pollution. Abbreviations: MCS—mosquito coil smoke; PTB—preterm birth.

## Data Availability

The data that support the findings of this study are available on request from the corresponding author. The data are not publicly available due to privacy or ethical restrictions.

## Supplementary Materials

The following supporting information can be downloaded at: https://www.mdpi.com/article/10.3390/ijerph191811771/s1, Figure S1: Flow chart of the analytic sample selection process; Figure S2: Directed acyclic graph (DAG) for the association between prenatal MCS exposure and PTB, showing all potential confounders. Pink lines indicate potential confounders; Table S1: Cross-over analysis for association between prenatal MCS exposure and PTB; Table S2: Cross-over analysis for association between prenatal MCS exposure and PTB, stratified by the child’s sex; Table S3: Associations between maternal MCS exposure during different periods of pregnancy and PTB (n = 61226); Table S4: Cross-over analysis for association between prenatal MCS exposure and PTB (n = 61226).
