# Supplementary material for: First Trimester of Pregnancy as the Sensitive Period for the Association between Prenatal Mosquito Coil Smoke Exposure and Preterm Birth
Source: Int J Environ Res Public Health. 2022 Sep 18;19(18):11771. doi: 10.3390/ijerph191811771 (PMC9517152; doi:10.3390/ijerph191811771)
Supplement: Supplementary file 1 [file ijerph-19-11771-s001.zip › ijerph-1872621-supplementary.pdf]

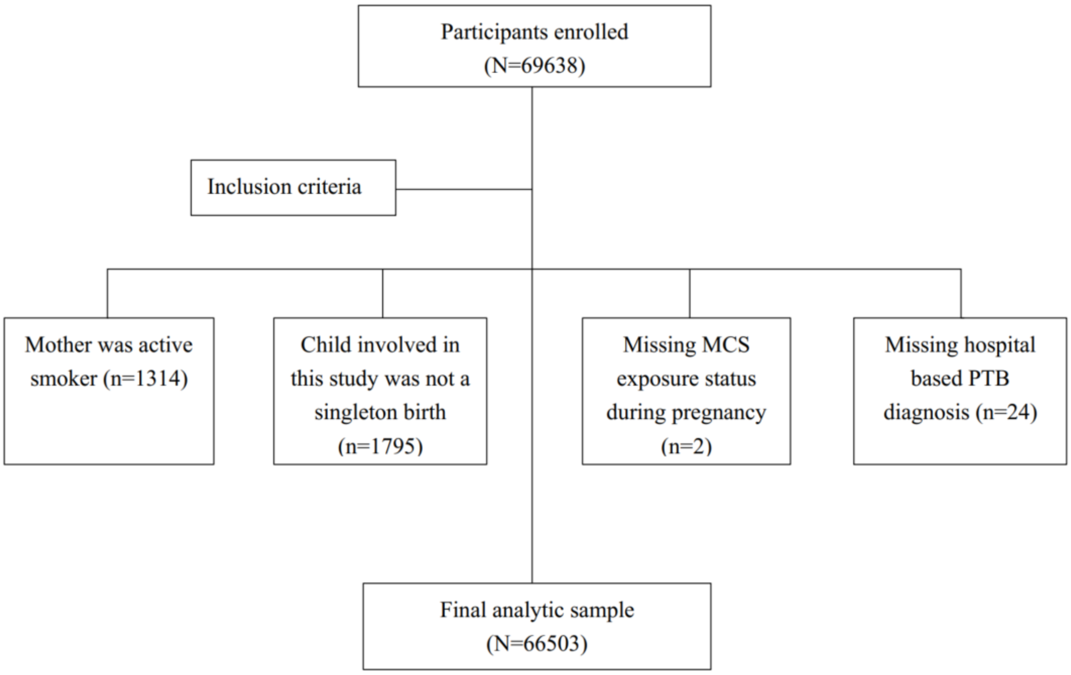

Figure S1. Flow chart of the analytic sample selection process.

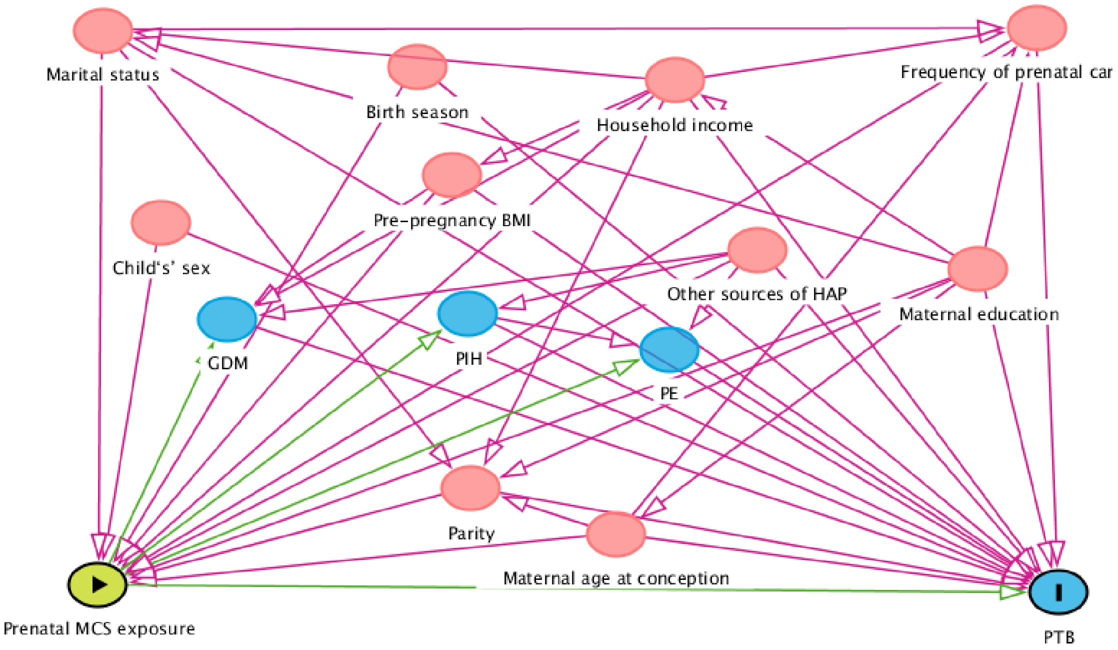

Figure S2. Directed acyclic graph (DAG) for the association between prenatal MCS exposure and PTB, showing all potential confounders. Pink lines indicate potential confounders.

MCS: mosquito coil smoke; PTB: preterm birth; BMI: body mass index; GDM: gestational diabetes mellitus; PIH: pregnancy induced hypertension; PE: Pre-eclampsia

Table S1. Cross-over analysis for association between prenatal MCS exposure and PTB.

MCS exposure			PTB/n	ORs (95% CI)	
The first trimester	The second trimester	The third trimester		Crude	Adjusted [a]
No	No	No	3186/46167	1.00	1.00
**Yes**	No	No	210/2411	1.29 (1.11, 1.49)	1.27 (1.09, 1.46)
No	**Yes**	No	34/549	0.89 (0.62, 1.24)	0.86 (0.60, 1.20)
No	No	**Yes**	47/781	0.86 (0.63, 1.15)	0.86 (0.63, 1.14)
**Yes**	**Yes**	No	118/1375	1.27 (1.04, 1.53)	1.23 (1.01, 1.48)
**Yes**	No	**Yes**	18/282	0.92 (0.55, 1.44)	0.90 (0.53, 1.40)
No	**Yes**	**Yes**	73/1146	0.92 (0.72, 1.16)	0.90 (0.70, 1.13)
**Yes**	**Yes**	**Yes**	1101/13792	1.17 (1.09, 1.26)	1.14 (1.06, 1.23)

[a] Adjustment for maternal age at conception, maternal education, marital status, household income, frequency of prenatal care visits, pre-pregnancy BMI, parity, child's sex, birth season, and exposure to other four sources of household air pollution.

Abbreviations: MCS, mosquito coil smoke; PTB, preterm birth

Table S2. Cross-over analysis for association between prenatal MCS exposure and PTB, stratified by the child's sex [#].

MCS exposure			aORs (95% CI)	
The first trimester	The second trimester	The third trimester	Boys	Girls
No	No	No	1.00	1.00
**Yes**	No	No	1.21 (0.99, 1.47)	1.34 (1.07, 1.66)
No	**Yes**	No	0.91 (0.57, 1.38)	0.78 (0.41, 1.33)
No	No	**Yes**	0.88 (0.58, 1.27)	0.83 (0.50, 1.29)
**Yes**	**Yes**	No	1.21 (0.93, 1.55)	1.24 (1.01, 1.49)
**Yes**	No	**Yes**	1.01 (0.53, 1.75)	0.73 (0.29, 1.53)
No	**Yes**	**Yes**	0.85 (0.61, 1.16)	0.96 (0.65, 1.35)
**Yes**	**Yes**	**Yes**	1.04 (0.94, 1.15)	1.27 (1.14, 1.42)

[#] Adjustment for maternal age at conception, maternal education, marital status, household income, frequency of prenatal care visits, pre-pregnancy BMI, parity, child's sex, birth season, and exposure to other four sources of household air pollution.

Abbreviations: MCS, mosquito coil smoke; PTB, preterm birth.

Table S3. Associations between maternal MCS exposure during different periods of pregnancy and PTB (n = 61226).

Exposure periods	MCS exposure	PTB/n	OR (95% CI)	
			Crude	Adjusted [a]
Entire pregnancy	No	2914/42599	1.00	1.00
	Yes	1429/18627	1.13 (1.06, 1.21)	1.11 (1.03, 1.18)
The first trimester	No	3053/44884	1.00	1.00

	Yes	1290/16342	1.17 (1.10, 1.26)	1.15 (1.07, 1.23)
The second trimester	No	3161/45801	1.00	1.00
	Yes	1182/15425	1.12 (1.04, 1.20)	1.09 (1.01, 1.17)
The third trimester	No	3240/46599	1.00	1.00
	Yes	1103/14627	1.09 (1.02, 1.17)	1.06 (0.99, 1.15)

[a] Adjustment for maternal age at conception, maternal education, marital status, household income, frequency of prenatal care visits, pre-pregnancy BMI, parity, child's sex, birth season, and exposure to other four sources of household air pollution.

Abbreviations: MCS, mosquito coil smoke; PTB, preterm birth.

Table S4. Trimester-specific association between prenatal MCS exposure and PTB (n = 61226).

MCS exposure			PTB/n	OR (95% CI)	
The first trimester	The second trimester	The third trimester		Crude	Adjusted [a]
No	No	No	2914/42599	1.00	1.00
**Yes**	No	No	185/2218	1.24 (1.06, 1.44)	1.22 (1.04, 1.42)
No	**Yes**	No	31/507	0.89 (0.60, 1.26)	0.86 (0.59, 1.22)
No	No	**Yes**	45/732	0.89 (0.65, 1.19)	0.89 (0.64, 1.19)
**Yes**	**Yes**	No	110/1275	1.29 (1.05, 1.56)	1.24 (1.01, 1.51)
**Yes**	No	**Yes**	17/252	0.99 (0.58, 1.56)	0.97 (0.57, 1.54)
No	**Yes**	**Yes**	63/1046	0.87 (0.67, 1.12)	0.86 (0.65, 1.10)
**Yes**	**Yes**	**Yes**	978/12597	1.15 (1.06, 1.24)	1.12 (1.03, 1.21)

[a] Adjustment for maternal age at conception, maternal education, marital status, household income, frequency of prenatal care visits, pre-pregnancy BMI, parity, child's sex, birth season, and exposure to other four sources of household air pollution.

Abbreviations: MCS, mosquito coil smoke; PTB, preterm birth.
